# The association between online class-related enjoyment and academic achievement of college students: a multi-chain mediating model

**DOI:** 10.1186/s40359-023-01390-1

**Published:** 2023-10-21

**Authors:** Youlai Zeng, Wenting Zhang, Jiaxin Wei, Wen Zhang

**Affiliations:** School of Education, Liaoning Normal University, 850 Huanghe Road, Dalian, 116029 People’s Republic of China

**Keywords:** Online class-related enjoyment, Learning engagement, School motivation, Academic achievement

## Abstract

**Background:**

Based on the control-value theory of achievement emotion and self-determination motivation theory, this study attempted to examine the multi-chain mediating relationships among online class-related enjoyment, school motivation, learning engagement and academic achievement.

**Methods:**

This is an empirical study based on cross-sectional data. Online class-related enjoyment is the independent variable, academic achievement is the dependent variable, and school motivation and learning engagement are the mediating variables. Sample data were collected from 1294 Chinese college students, and SPSS macro program PROCESS 3.3 was used for data analysis.

**Results:**

The present study confirmed that students’ online class-related enjoyment has a significant positive correlation with academic achievement. And there is a positive correlation between college students’ school motivation with learning engagement and college students’ learning engagement with academic achievement. In addition, online class-related enjoyment affects academic achievement through the chain mediating effect of school motivation and learning engagement.

**Conclusions:**

Our study indicated that online class-related enjoyment has a significant impact on academic achievement. Both of these factors should be considered when determining the optimal multi-chain mediating model for Online Class-related Enjoyment and Academic Achievement of college students.

## Introduction

Under the background of the digital age, “Internet + education” conforms to the changing trend of technology-driven teaching and is constantly constructing a new education ecology. Online learning is an activity that takes learners as the principal role to participate in the practice. The emotional experience of learners in the online learning environment is essential, which may impact the process and results of learners’ online learning. Therefore, this study focused on college students’ emotional experience of online-related enjoyment. It empirically explores the relationship between online-related enjoyment and academic achievement to provide references for improving the effectiveness of online class-related teaching.

### Online class-related enjoyment

Emotions include specific emotional, psychological, and behavioral elements [1, 2]. Emotions are ubiquitous in academic settings and involved in virtually every aspect of the teaching and learning process [3]. With the advance and development of technology tools, online learning has appeared as one of many explanations and terminology for digital learning [4]. Like other forms of learning, online learning is full of emotional experience [5], learners may have a negative emotional experience of frustration or despair or a positive emotional experience of joy or pleasure. Pekrun proposed the concept of academic emotion for the first time, believing that academic emotion is the emotion directly related to school learning, teaching, and academic achievements, such as excitement in the learning process, pride in success, and anxiety related to exams [6]. Enjoyment, as one of the positive emotions, has attracted the attention of many scholars [7–9]. Enjoyment is defined as the sense of satisfaction and reward derived from the activity or the outcome of the activity [10]. Enjoyment of learning is regarded as an individual tendency to respond to a specific situation with a specific level of enjoyment in a learning environment [11], and is an activity-related, activating, positive emotion [12, 13]. According to the control-value theory of achievement emotion proposed by Pekrun [14], online class-related enjoyment was defined from three dimensions including valence (positive and negative), activity (arousal), and target orientation (arousal object). It is assumed that online learning and related materials have a positive value, and the activity is perceived as completely self-controlled.

### Learning engagement

Learning engagement is an essential aspect of online learning [15, 16]. Kuh formally proposed the concept of college students’ engagement, which first introduced the concept of engagement in learning in higher education [17]. It was further promoted and disseminated in the National Survey of Learning Engagement [18]. Different scholars had different definitions of the connotation of learning engagement. Some scholars believed that learning engagement includes behavior and emotion, defined as student-initiated learning behaviors, efforts, academic task persistence, and emotional states in the learning process [19]. Other scholars believed that learning engagement includes academic engagement and social engagement [20]. With the deepening of research, scholars gradually accepted the three-dimensional division of learning engagement. Schaufeli constructed a three-dimensional work engagement model and introduced the study of work engagement in student groups, believing that learning engagement is a positive and fulfilling mental state related to learning, including three dimensions of vitality, dedication, and concentration [21]. Fredricks believed that learning engagement should consist of three dimensions, such as behavioral engagement, cognitive engagement, and emotional engagement [22]. This study draws on the definition of Fredricks, which defines learning engagement as students’ involvement in their learning activities, regarding behavior (participation in academic and class-related activities, attention, engagement, concentration, completing assignments, and following class rules), emotion (positive emotion towards teachers, classmates, class activities, interests, hobbies, and identification with the school or subject area) and cognition (commitment to learning, self-regulation, persistence, and the effort to understand complex ideas or master difficult skills).

### School motivation

School motivation refers to the motivation for individual students to make some learning decisions, participate in activities and persist in pursuing demanding learning processes. The reasons why students participate in learning are usually explained as intrinsic motivation, which is the behavior of experiencing the happiness and satisfaction of the activity itself, and extrinsic motivation, which is the behavior of achieving some goals, such as obtaining rewards and avoiding punishments [23]. When students learn out of intrinsic motivation, they perceive learning as rewarding. In contrast, when students learn out of extrinsic motivation, they perceive learning activities as achieving a desired outcome. Studies have confirmed that intrinsic motivation is correlated with higher engagement and academic performance [24], and extrinsic motivation is also significantly correlated with engagement in learning [25]. Intrinsic motivation is considered a sustainable participatory motivation in learning [26]. Based on goal theory, McInerney believed that school motivation comprises complex motivational goals that are interrelated and developed the Inventory of School Motivation including a total of eight first-order factors (task, effort, competition, social power, affiliation, social concern, praise, and token), four second-order factors (mastery, performance, social factors, and extrinsic factors) [27, 28]. This scale has been widely concerned and cited in the academic community [29–31]. The mastery goal dimension of tasks and efforts belongs to the internal school motivation, while the external factor dimension composed of rewards and praise is the external school motivation.

### The relationship between online class-related enjoyment and school motivation

In the self-determination theory of Deci and Ryan, intrinsic motivation was described as a commitment to a task arising from interest and affection for the task itself. When individuals viscerally recognize the value of academic tasks, they commit to them wholeheartedly and consistently [32]. Intrinsic motivation in the online learning context shows the learner’s interest in gaining new knowledge and skills to grow in his/her field [33]. For example, the students engage in online learning who enjoy learning and are interested in subjects. Extrinsic motivation refers to undertaking tasks because of instrumental reasons. The learner becomes extrinsically motivated through good grades, awards, and prizes. The learner’s motivation for learning directs his/her efforts towards his/her learning desires, rehearsal, retention, and retrieval [34]. For example, students are motivated to pass an exam to earn grades, appreciation, and/or avoid punishment.

Based on the control-value theory, Pekrun et al. revealed that positive academic emotions improve school motivation [6]. And Pekrun defined intrinsic motivation as any motivation from the behavior itself, whose existence is promoted by positive emotions and extrinsic motivation regarding the motivation of the outcome of the task action, and all the emotions related to the outcome will have an impact on the extrinsic motivation [35].A highly intrinsically motivated person, driven by an emotional state that something is fun to do, pursues an operation and does it for its sheer intrinsic pleasure and enjoyment [36]. Learners who perceive online learning as enjoyable and rewarding are more intrinsically motivated and optimistic about the course. As a result, with the support of intrinsic and extrinsic motivation, they may be filled with enjoyment and energy while attending online courses, increasing the positive emotions and cognition of online learning interactions [37].

## Hypothesis 1:   Online class-related enjoyment of college students is significantly positively correlated with their school motivation

### The relationship between school motivation and learning engagement

Firstly, school motivation and learning engagement are a pair of related concepts. Learning engagement concerns the quality and quantity of student involvement, or connections to educational endeavors and thus to the activities, values, individuals, goals, and places that make up education. Studies of motivation and engagement also tend to be intertwined [38]. Secondly, an important finding of this study is that school motivation impacts learning engagement [39] ,and school motivation is an antecedent of learning engagement [40]. Individuals with high school motivation are shown to be focused on solving problems, full of vitality in activities in class, and willing to devote their time and energy to learning, and this continuous and positive enthusiasm for learning provides an important mechanism. Through this mechanism, individuals can ensure focus, vitality, and dedication to learning, namely learning engagement. Finally, the stronger the regulation ability of students’ school motivation, the stronger their learning adaptability, and the more pronounced the influence on learning engagement [41]. Some scholars even believed that learning engagement can be regarded as an external expression of school motivation [38]. According to the self-system model based on motivational development [32, 42], and a model of factors influencing learning engagement [22, 43], there was a significant correlation between context, self, action, and outcome. This study concludes that college students’ school motivation is closely related to learning engagement.

## Hypothesis 2:  A significant positive correlation exists between college students’ school motivation and learning engagement

### The relationship between learning engagement and academic achievement

Academic achievement has long been considered an important outcome of learning engagement. In existing research, scholars agreed that highly engaged students were effective learners and that learning engagement positively impacted academic performance [19]. Students’ participation in meaningful learning activities can promote the formation of their thinking habits and enhance their ability to continue learning and personal development [44]. Thus, engagement in learning can lead to positive academic achievement and learning outcomes. Usually, students with behavioural engagement are active, have positive attitudes, and are able to self-regulate their learning, put in a high amount of effort, and participate in every learning task [45]. King concluded that academic performance positively correlates with behavioral and emotional engagement [46]. Pietarinen found a positive correlation between cognitive engagement and academic achievement [47]. High student participation promotes academic success [48], further promoting students’ participation in learning activities, thus forming a virtuous cycle of learning [49]. The meta-analysis results showed that the average effect size of the correlation between learning engagement and academic achievement was higher than cognitive engagement and emotional engagement [50]. Learning engagement and academic achievement are essential in an online-learning environment [51]. Similarly, previous studies have also confirmed that there is a significant relationship between learning engagement in online-learning environments and their academic achievement [52]. In short, students’ behaviour engagement can determine the success of their learning, particularly in an online setting [53]. In all, the positive effect of learning engagement has been verified in many studies. Therefore, this study believes that the higher the degree of learning engagement of college students, the higher the level of academic achievement.

## Hypothesis 3:  A significant positive correlation exists between learning engagement and academic achievement

### The relationship between online class-related enjoyment, school motivation, learning engagement and academic achievement

Positive academic emotions can promote learners’ cognition (such as self-regulation), motivation, and behavior (such as learning strategies and engagement), which further facilitate academic achievement [12]. There are also emotional elements in online learning settings. Existing studies have confirmed that adult distance learners will produce various emotional states during online learning [54, 55]. And some studies have found that social interaction can alleviate the loneliness of learners during online learning, increase the positive emotional experience, maintain learners’ motivation for continuous learning, and improve learners’ willingness and involvement [56, 57]. At the same time, learning engagement is an effective way for students’ motivational process to guide individual learning outcomes and ability growth [58]. Positive and highly aroused emotions predict using deep learning strategies, thus increasing learners’ effort and learning involvement [59]. In other words, students with high engagement will focus on learning, attend classes on time, observe classroom discipline, pay more attention, persevere when encountering difficulties, and use effective learning strategies. Online class-related enjoyment, as a positive academic emotion, is also in a state of mutual influence with learners’ learning involvement in online learning.

Self-determination Theory (SDT) is a theory of motivation in essence, focusing on the the motivational process of individual self-determined behavior and highlighting the active role of individual in the motivation process [32, 60, 61]. Self-determination Theory provides an important theoretical lens for understanding the relationship between online class-ralated enjoyment and school motivation. One of the core principles of SDT is emotion, which provides information that can lead to formation of motivation and subsequent purposeful behavior [62]. Self-determined behavior is powered by motivation, which is caused by emotions and the need to satisfy the state in the future. Some scholars have found that high engagement in learning is a sign of students’ active pursuit of academic progress and plays a crucial role in academic achievement [22]. In addition, SDT theory holds that satisfying basic psychological needs can stimulate learners’ motivation and promote learning engagement [63]. The satisfaction of basic psychological needs promotes the intrinsic school motivation. Individuals driven by intrinsic motivation will perform better academically [64, 65]. Research has shown that students’ enjoyment of learning materials can promote more participation in learning activities and better learning outcomes [7, 66]. Students’ enjoyment of learning can also improve their expected performance, thus strengthening and motivating them to devote themselves to learning [67]. Therefore according to the SDT, the present study suggests that school motivation and learning engagement mediate the influence of online class-related enjoyment on academic achievement.

## Hypothesis 4:  School motivation and learning engagement play a chain mediating role in the influence of online class-related enjoyment on academic achievement

### Research methods

#### Research design

**Fig. 1:**
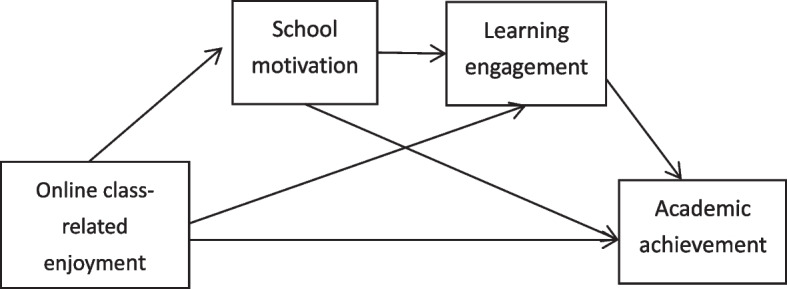
Research overall model diagram

This is an empirical study based on cross-sectional data. Online class-related enjoyment is the independent variable, academic achievement is the dependent variable, and school motivation and learning engagement are the mediating variables. The research model is shown in Fig. 1.

#### Participants

Some universities were selected as sample schools in the eastern, central, and western regions of China, including Dongbei University of Finance and Economics, Northeastern University, Dalian Medical University, Northeast Petroleum University, Gannan Normal University, Shaoxing University, Liaoning Normal University, etc. A total of 1294 undergraduates (368 male, 926 female) were randomly selected as research objects. In addition, given the difference in the application of online classes to different college majors, all majors of humanities and social sciences (658 students) and science and technology (636 students) were covered in the selection of schools. In the grade distribution, the freshman 394 students, sophomores 445 students, juniors 354 students, and seniors 101 students. At the same time, college students judged the academic ranking of their majors, including 177 students in the top 20%, 325 students in the 21–40%, 500 students in the 41–60%, 214 students in the 61–80% and 78 students in the last 20%.

#### Measurements

##### Online class-related enjoyment

The Achievement Emotion Questionnaire (AEQ) is a multi-dimensional self-report tool designed to assess the achievement emotion of college students and examine the emotion experienced by students in the context of academic achievement [6]. AEQ can assess class, study, and test-related emotions such as enjoyment, hope, pride, anger, anxiety, shame, despair, and boredom. This study uses the class-related enjoyment scale, which includes nine questions. It adjusts some statements according to the online class-related achievement context, such as changing “classroom” to “online classroom”. For example, “I am looking forward to learning a lot in online class.” “I enjoy being in the online class.” The five-point Likert scale was used to judge from “completely disagree” to “strongly agree”. Cronbach’s alpha verified by Pekrun was 0.85. The internal consistency reliability coefficient in this study is 0.90.

##### School motivation

The Inventory of School Motivation compiled by Mcinerney and Ali includes eight first-order factors, which can be divided into task, effort, competition, social power, affiliation, social concern, praise, and token, and four second-order factors, including mastery, performance, social factors, and extrinsic factors [27]. In this study, mastery and extrinsic factors from Mcinerney’s Inventory of School Motivation were selected using translation-back translation method. A graduate student who passed TEM-8 translated English into Chinese, and then a graduate student majoring in English translated back the items into English. The Chinese version of the scale was finally formed, with a total of 23 items. For example, “I try harder with interesting work”, and “I work hard in class for rewards from the teacher”. Task and effort represent intrinsic school motivation and praise and token represent extrinsic school motivation. Mcinerney surveyed 697 people in China and tested the cross-cultural reliability and validity of the questionnaire. The results showed that Cronbach’s alpha of four-factor dimensions (Task, effort, praise, and token) was 0.55, 0.70, 0.77, and 0.72, respectively. Cronbach’s alpha of each factor dimension in this study was 0.92, 0.93, 0.87, and 0.83, respectively.

##### Learning engagement

In this study, the definition of learning engagement is referenced by Fredricks (2004) [22]. Learning engagement is understood as students’ investment in their learning activities, including behavior, cognition, and emotion. The questionnaire consisted of 19 questions, including three inverse questions, rated on a five-point Likert scale from “completely disagree” to “strongly agree.“ In this study, the “translation-back translation” method is adopted to ensure the accuracy of item translation. For example, “I finished my assignment on time.“ “I feel happy in school.“ “I will study hard even if there are no exams.“ The internal consistency reliability coefficient of each dimension is between 0.72 and 0.86 in Fredricks’ study. The internal consistency reliability coefficient in this study is 0.96.

##### Academic achievement

Following the practice of scholars such as Lizzio et al. [68], the academic achievement variable is represented comprehensively by general skills. In this study, generic skills mainly measure skills directly related to employment and lifelong learning, such as written communication, problem-solving, analytical skills, teamwork, and self-management. For example, “Problem-solving skills improved” and “working collaboratively as a team member.“ The academic achievement questionnaire included seven questions in total, and Cronbach’s alpha of this part was 0.96.

#### Data analysis

All analysis was conducted using SPSS 25.0 and AMOS 23.0. Pearson correlation statistics were used to establish the relationships between the study variables. Four hypotheses were tested through SEM.

## Results

### Descriptive statistics and correlation analysis

The descriptive statistics and correlation analysis results of variables in this study are shown in Table 1. There was a significant positive correlation between online class-related enjoyment and academic achievement (*r* = 0.712, *p* < 0.01). School motivation was positively correlated with academic achievement (*r* = 0.735, *p* < 0.01). There was a significant positive correlation between learning engagement and academic achievement (*r* = 0.757, *p* < 0.01).

**Table 1:** Mean, standard deviation, correlations

Variables	M	SD	1	2	3	4
1.OCE	3.449	0.611	1			
2.SM	3.448	0.595	0.712^a^	1		
3.LE	3.492	0.601	0.791^a^	0.803^a^	1	
4.AA	3.482	0.739	0.712^a^	0.735^a^	0.757^a^	1

*OCE* Online class-related enjoyment, *SM* School motivation, *LE* Learning engagement, *AA* Academic achievement

^a^Correlation is significant at the 0.01 level (2-tailed)

### Chain mediation analysis

**Fig. 2:**
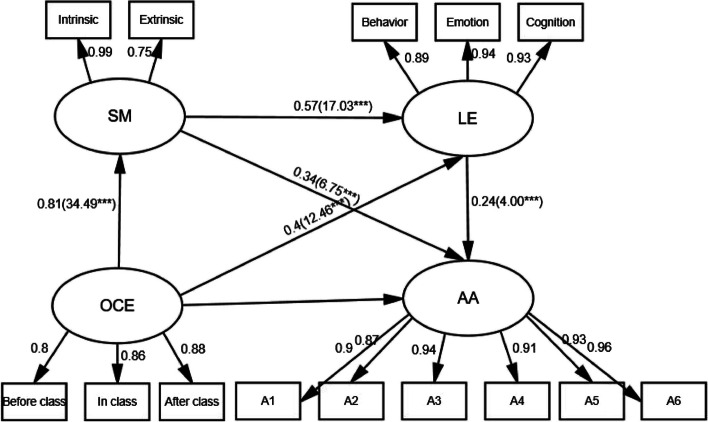
Schematic diagram of chain mediation. Note: All figures in the model are completely standard; ***. Correlation is significant at the 0.001 level (2-tailed)

According to the assumptions of the model, the structural equation is established. Model fit index (χ^2^/ df = 6.676, RMSEA = 0.066, CFI = 0.984, TLI = 0.976, SRMR = 0.023) basically meet the recommended standards. Figure 2 illustrates the standardized load of observed variables on each latent variable and the path coefficient between variables, and the coefficient index of standardized equation is shown in Table 2. The results showed that online class-related enjoyment was significantly positively correlated with school motivation (β = 0.805, *p* < 0.001), and hypothesis 1 was confirmed. There was a significant positive correlation between online class-related enjoyment and learning engagement (β = 0.401, *p* < 0.001), hypothesis 2 was confirmed. There was a significant positive correlation between learning engagement and academic achievement (β = 0.242, *p* < 0.001), and hypothesis 3 was confirmed.

**Table 2:** Index of coefficient of standardized equation

Outcome variable	Predictive variable	R^2^	β	SEs	t	p	LLCI	ULCI
Equation 1								
SM	OCE	0.649	0.805	0.096	34.488	***	0.753	0.849
Equation 2								
LE	OCE	0.858	0.401	0.049	12.464	***	0.306	0.498
	SM		0.572	0.012	17.034	***	0.476	0.664
Equation 3								
AA	OCE	0.647	0.263	0.019	6.187	***	0.147	0.388
	SM		0.341	0.005	6.75	***	0.191	0.505
	LE		0.242	0.017	3.999	***	0.079	0.40

*OCE* Online class-related enjoyment, *SM* School motivation, *LE* Learning engagement, *AA* Academic achievement

***. Correlation is significant at the 0.001 level (2-tailed)

The Bootstrap method (repeated sampling 5000 times) was used to analyze the chain mediation effect test in Table 3. Online class-related enjoyment has an impact on academic achievement through the partial mediating effect of school motivation and learning engagement. The mediating effect value of school motivation is.275, and that of learning engagement is.097, respectively accounting for 36.8% and 13.0% of the total effect of online class-related enjoyment on academic achievement (0.747). The effect value of the chain mediating effect between school motivation and learning engagement was.112, accounting for 15.0% of the total effect.

**Table 3:** Tests the mediation effect based on bootstrap method

Effect types	Effect	Boot SE	Boot LLC	Boot ULC	Ration of indirect to total effect
Total effect	0.747	0.022	0.701	0.787	
Direct effect	0.263	0.061	0.147	0.388	
Total indirect effect	0.484	0.051	0.384	0.585	64.8%
OCE—>SM—>AA	0.275	0.066	0.154	0.416	36.8%
OCE—>LE—>AA	0.097	0.035	0.034	0.173	13.0%
OCE—>SM—>LE—>AA	0.112	0.04	0.039	0.193	15.0%

*OCE* Online class-related enjoyment, *SM* School motivation, *LE* Learning engagement, *AA* Academic achievement

Using group analysis of structural equation model to explore group differences by gender, major, and grade, the constrained model estimation is carried out with the paths of each group model set to be equal. The significance of the moderated effect was determined by Chi-square difference test between unconstrained model and constrained model. The results showed that the restriction of equal structural weight between male and female groups had a significant effect on the model (Δχ2 (6) = 14.564, *p* < 05), but there were no significant effect on model in different professional groups (Δχ2 (6) = 10.427, p > 0.05) and grade groups (Δχ2 (18) = 37.060, p > 0.05). All groups are estimated with unconstrained model and standardized regression weights for gender in unconstrained model showed in Table 4.

**Table 4:** Unconstrained model standardized regression weights for gender

Path	Male	Female
OCE—>SM	0.845**	0.786**
OCE—>LE	0.337**	0.424**
SM—>LE	0.64***	0.549***
OCE—>AA	0.226*	0.271**
LE—>AA	-0.012	0.313**
SM—>AA	0.675**	0.235**

*Correlation is significant at the 0.05 level (2-tailed); **Correlation is significant at the 0.01 level (2-tailed); ***Correlation is significant at the 0.001 level (2-tailed)

The mediating effect test results of male and female group are shown in Table 4. In male group, online class-related enjoyment has an impact on academic achievement through the mediating effect of school motivation, and the mediating effect value is 0.571, accounting for 72.65% of the total effect of Online class-related enjoyment on academic achievement (0.786). In female group, online class-related enjoyment has an impact on academic achievement through the mediating effect of school motivation and learning engagement, and the mediating effect value is 0.452, accounting for 62.52% of the total effect (0.723). The results show that the mediating effect of students’ online class-related enjoyment on academic achievement through school motivation and learning engagement is moderated by gender in Table 5.

**Table 5:** The mediating effect test of male and female group

Group	Path	Indirect effect	Relative mediating effect	Boot LLC	Boot ULC
Male	OCE—>SM—>AA	0.571	72.65%	0.284	1.031
	OCE—>LE—>AA	-0.004		-0.129	0.118
	OCE—>SM—>LE—>AA	-0.006		-0.289	0.171
Female	OCE—>SM—>AA	0.184	25.21%	0.062	0.317
	OCE—>LE—>AA	0.133	18.40%	0.058	0.224
	OCE—>SM—>LE—>AA	0.135	18.67%	0.056	0.232

## Discussion

### The significant positive correlation between online class-related enjoyment and academic achievement

The study confirmed that online class-related enjoyment had a significant positive correlation with academic achievement. This is consistent with existing research results. For instance, some scholars have found that positive emotional experience plays a vital role in achievement and significantly impacts students’ academic success [69]. Pekrun used longitudinal research data and tested structural equation models to show that positive emotions (enjoyment and pride) positively predicted final math exam scores, confirming the importance of emotions on students’ achievement [70]. Similarly, Camacho-Morles demonstrated a positive correlation between enjoyment and student achievement [71]. Students’ enjoyment has been demonstrated as playing an essential role in promoting learners’ academic achievement and personal growth [72]. Specifically, enjoyment positively predicted academic achievement [73]. Positive emotions can contribute to the decrease in the effects of negative emotional arousal and facilitate adaptation, as the online learning offers more opportunities for emotions regulation [74, 75]. In other words, online class-related enjoyment acts as a protective factor in online learning [76]. The results of this study further support the hypothesis of the control-value theory of achievement emotion. According to the control-value theory of achievement emotion, positive high-arousal emotions such as the joy of learning benefit academic achievement under most conditions in online learning.

###  The mediating role of school motivation

The study found that school motivation mediated the effect of online class-related enjoyment on academic achievement. This is consistent with the conclusion of existing studies. On the one hand, studies have shown that positive emotions are significantly related to motivation. Emotions are powerful sources of information that influence motivational patterns [77, 78], not just the variables of motivation generation [79]. Promoting positive emotions can positively influence the interdependence between emotion, motivation, and cognition [80]. Intrinsic motivation and extrinsic motivation were positively inter-related, and significant associations between intrinsic motivation and enjoyment, and willingness were consistent with the SDT that students who are passionate about the subject tend to enjoy studying and be more willing to study without external rewards [81]. On the other hand, existing studies have proved that motivation is significantly correlated with students’ academic achievement [82, 83]. Both intrinsic and extrinsic motivation act as a mediating role for academic performance through online learning behaviours [84]. And intrinsic motivation was significantly and positively associated with self-efficacy, learning engagement, and academic performance in medical students [85]. Therefore, the stimulation of school motivation plays an important role in the influence of online class-related enjoyment on academic achievement and provides important operational ideas for teaching practice.

### The mediating role of learning engagement

The study found that learning engagement mediates the effect of online class-related enjoyment on academic achievement. Existing studies have shown that positive emotions can make learners feel a good sense of mastery, and learners are more willing to construct knowledge actively and have a higher degree of learning involvement [86]. Other research results show that engagement is positively correlated with more active learning activities [87, 88], and engagement is significantly correlated with academic achievement [89]. In online learning, efficient social interaction promotes learners to experience more positive emotions, more positive learning experiences, and increased learning engagement [90]. Positive emotions in online learning promote the use of deep-processing learning strategies, such as critical thinking about learning content, which predict higher levels of learning engagement [91]. Students with a positive emotional experience of learning content and materials can obtain higher academic performance and emotional involvement is indirectly related to learning achievement [92]. Learning engagement in the learning process increases when students find study engaging, effective, and valuable in online learning [81]. To sum up, it can be found that learning engagement can promote students’ academic efforts and persistence, help students experience their intrinsic learning interests, and achieve excellent academic performance. Online class-related enjoyment directly affects academic achievement and indirectly affects academic achievement through learning engagement.

### Online class-related enjoyment affects academic achievement through the chain mediating effect of school motivation and learning engagement

This study confirmed that school motivation and engagement mediate the correlation between online class-related enjoyment and academic achievement. Zhou argued that the influence of emotion on achievement is indirect and can be realized through cognitive processes, motivational mechanisms, and interpersonal resources [93]. Valiente suggested that researchers should consider the mediating role of cognitive processes and motivational mechanisms in the relationship between emotion and academic achievement [94]. In this regard, numerous studies later showed that achievement emotion affects learning achievement by influencing the regulation process of cognition and motivation, such as school motivation and learning strategies [95]. Emotions of online learners are significant [96, 97], and the positive emotion promoting learning process is experienced by online learners [54, 98, 99]. On the contrary, when students get good grades, they will have a high sense of control value [100, 101], and then generate positive emotional experiences. Therefore, the influence of positive emotions on learning is manifested as stimulating students’ school motivation, improving their learning efficiency and commitment, and thus helping students achieve better learning outcomes [102]. SDT divides motivation into non-motivation, intrinsic motivation and extrinsic motivation [63]. This study examines the motivation types of self-determination theory from the perspective of empirical research, including intrinsic and extrinsic school motivation. Compared with other scholars who only test SDT from the perspective of intrinsic motivation [64], the present study has certain theoretical expansion value and reference value for future scholars’ research.

### Research limitations and directions

First of all, in terms of sample selection, due to the limitation of sampling conditions, the number of universities is not large enough, and the college level cannot cover all types. Therefore, future studies may consider expanding the sample coverage to further test the findings. Secondly, cross-sectional data is used to analyze the relationship between factors, and other factors related to online classes are not controlled enough, and quasi-experimental research can be tried in future research. Third, in terms of measurement tools, the online class-related enjoyment scale uses part of Pekrun’s achievement emotion questionnaire, and it is necessary to develop a special survey tool to measure online class-related enjoyment in future research. Finally, the mechanism of online class-related enjoyment stimulating school motivation and promoting learning engagement of college students is still unclear, so we can explore further in the future to provide a more detailed influence mechanism and decision-making basis for educational practice.

## Conclusion and research contribution

In the context researched, using the self-determination theory and the control-value theory of achievement emotions as a framework, our study identified that college students’ online class-related enjoyment had a significant positive correlation with academic achievement and it is mediated by school motivation and learning engagement. Specifically, college students’ school motivation has a positive correlation with learning engagement that also has a positive correlation with academic achievement. Online class-related enjoyment affects academic achievement through the chain mediating effect of school motivation and learning engagement. In terms of research significance, on the one hand, it suggests that college teachers should pay attention to the positive emotion, school motivation and learning engagement of college students in online classroom in order to improve teaching practice. On the other hand, we further empirically test the self-determination theory.

From the perspective of teaching practice, on the one hand, teachers should pay attention to students’ enjoyment experience in online classroom. The enjoyment experienced by college students in online learning can stimulate their desire and interest in learning and encourage them to participate in learning interaction, thus improving their academic achievement, and promoting their academic success, which also indicates that school motivation and learning engagement have a synergistic effect on academic achievement. Therefore, teachers should prepare lessons carefully in online classroom teaching, enhance the class interest, and pay attention to the student-centered education concept. So that students can experience a pleasant classroom atmosphere. On the other hand, teachers should pay attention to the use of teaching strategies to enhance students’ school motivation. Students with high school motivation are usually more willing to maintain focus, vitality, and dedication in learning activities, that is, a high level of learning engagement. This study further found that the mediating effect value of school motivation (0.275) was larger than that of learning engagement (0.097), indicating that the promoting effect of school motivation was stronger than that of learning engagement. Therefore, while improving the enjoyment of college students in online classes, we should try our best to promote the participation and interaction of students, especially the stimulation of students’ school motivation, to help students achieve academic success.

## Data Availability

The data that support the findings of this study are available upon request from the corresponding author.
